# Balance and Posture in Children and Adolescents: A Cross-Sectional Study

**DOI:** 10.3390/s22134973

**Published:** 2022-06-30

**Authors:** Nelson Azevedo, José Carlos Ribeiro, Leandro Machado

**Affiliations:** 1 CICS, ISAVE, Faculdade de Desporto da Universidade do Porto, 4200-450 Porto, Portugal; nelson.azevedo@docente.isave.pt; 2 CIAFEL, ITR, Faculdade de Desporto da Universidade do Porto, 4200-450 Porto, Portugal; jribeiro@fade.up.pt; 3 CIFI2D, LABIOMEP, Faculdade de Desporto da Universidade do Porto, 4200-450 Porto, Portugal

**Keywords:** children, adolescents, posture, balance, back pain

## Abstract

Balance and posture are two topics that have been extensively studied, although with some conflicting findings. Therefore, the aim of this work is to analyze the relationship between the postural angles of the spine in the sagittal plane and the stable static balance. A cross-sectional study was conducted with children and adolescents from schools in northern Portugal in 2019. An online questionnaire was used to characterize the sample and analyze back pain. Spinal postural angle assessment (pelvic, lumbar, and thoracic) was performed using the Spinal Mouse^®^, while stabilometry assessment was performed using Namrol^®^ Podoprint^®^. Statistical significance was set as α = 0.05. The results showed that girls have better balance variables. There is a weak correlation between the anthropometric variables with stabilometry variables and the postural angles. This correlation is mostly negative, except for the thoracic spine with anthropometric variables and the lumbar spine with BMI. The results showed that postural angles of the spine are poor predictors of the stabilometric variables. Concerning back pain, increasing the postural angle of the thoracic spine increases the odds ratio of manifestation of back pain by 3%.

## 1. Introduction

There are several definitions of good posture [1,2], but Kendal et al. have presented a definition that we found interesting: “good posture is that state of muscular and skeletal balance which protects the supporting structures of the body against the injury or progressive deformity, irrespective of the attitude (erect, lying, squatting or stooping) in which these structures are working or resting. Under such conditions, the muscles will function most efficiently, and the optimum positions are afforded for the thoracic and abdominal organs” [3]. Posture cannot be considered only as a static reflex response but is rather a complex competence based on the interaction of sensory-motor processes.

The effects of postural changes on health are not limited to adults but are also present in children. These effects are increasingly well described in the literature, and there is evidence of associated risk factors [4].

Understanding the relationship between posture and balance in children and adolescents is becoming increasingly important today due to lifestyle changes and their interrelationship with other musculoskeletal pathologies [5]. 

Balance involves the coordination of sensorimotor strategies to stabilize the body’s center of pressure (CoP) in the presence of both self-initiated and externally-initiated disturbances of stability [6]. Balance control can be defined as the appropriate response to perturbations of the center of pressure caused by the oscillation of the center of gravity, motor activity, or conscious interaction with the environment [7].

Balance can be divided into four types, namely: stable static balance (i.e., maintaining a stable position while standing), stable dynamic balance (i.e., maintaining a stable position while walking), proactive balance (i.e., anticipating a predicted balance disturbance), and reactive balance (i.e., compensating for an unforeseen balance disturbance) [8].

However, when we talk about balance and its relationship with gravity, we must necessarily talk about the foot. The foot contributes to the maintenance of postural stability by providing mechanical support to the body through the arch of the foot, among other structures, and coordinated coactivation of the lower limb muscles, as well as sensory information about body position and proprioception of the plantar cutaneous mechanoreceptors [9]. The importance of the foot and its relationship to the spine and back pain is becoming increasingly important [10]. 

For efficient balance control, it is necessary for the spine to have postural competence.

Spinal postural competence can be defined as the equilibrium between the external forces acting on the spine and the muscular response of the trunk, which is sensory regulated to maintain a stable upright posture, both static and dynamic [11,12]. Therefore, the relationship between the foot to provide sensory information and the spine is critical for optimal posture and efficient balance control, both in adults and children [13].

Recent studies have not found a direct relationship between children’s posture and balance disorders [14,15]. Ludwig et al. [15] suggested that balance and posture are complex interdependent mechanisms that should be better studied and understood. The study by Zurawski et al. [13] found a relationship between posture and balance in children and adolescents. Posture is also related to the occurrence of back pain in children, and it is considered a triggering risk factor [16,17].

Several studies included in a review article associate manifestations of back pain in adult subjects with balance deficits assessed by CoP stability parameters measured with pressure platforms [18]. 

From all these literature results, it becomes apparent that the relationship between posture and balance is an important topic for study and deeper understanding in children.

With the present study, we aim to deepen the understanding of the relationship between children’s and adolescents’ balance and changes in their posture in the sagittal plane.

To our knowledge, there is no study that examines the relationship between balance and posture using the pressure platform and the Spinal Mouse. By linking these two assessment tools, we expect to further explore the relationship between balance and posture.

### Hypotheses

**Hypotheses** **1.** *There is a relationship between postural angles in the spine regions with stable static balance in children and adolescents.*

**Hypotheses** **2.** *There is an association between postural angles in the spine regions and stable static balance with the manifestation of back pain in children and adolescents.*

## 2. Materials and Methods

A cross-sectional study was carried out with children and adolescents from schools in the north of Portugal, in the district of Braga, between October and December 2019, comprising the beginning of the school year.

A population analysis was performed to calculate the sample size. In 2019, the number of students enrolled from the 5th to the 12th grade in mainland Portugal was 576,436 [19]. With this population, the minimum size required for our study was 1066, with a margin of error of 3% and a confidence interval of 95% [20]. The study proposal was presented to the school director as well as to the physical education department. The benefits and potential risks of the study were explained. After approval of the study, we provided all children and adolescents in the school cluster with a description of the study and the informed consent form. All participants had the opportunity to participate or withdraw. After a period of analysis by the parents and legal guardians of the children, in which it was possible to clarify all doubts and questions related to the study, namely the benefits/risks, we obtained the written informed consent of all parents and guardians of the children involved in the study. The adults who participated in the study also signed the written informed consent.

Exclusion criteria were defined as participants who had musculoskeletal deficiencies or serious medical conditions that made data collection difficult or impossible.

**Figure 1:**
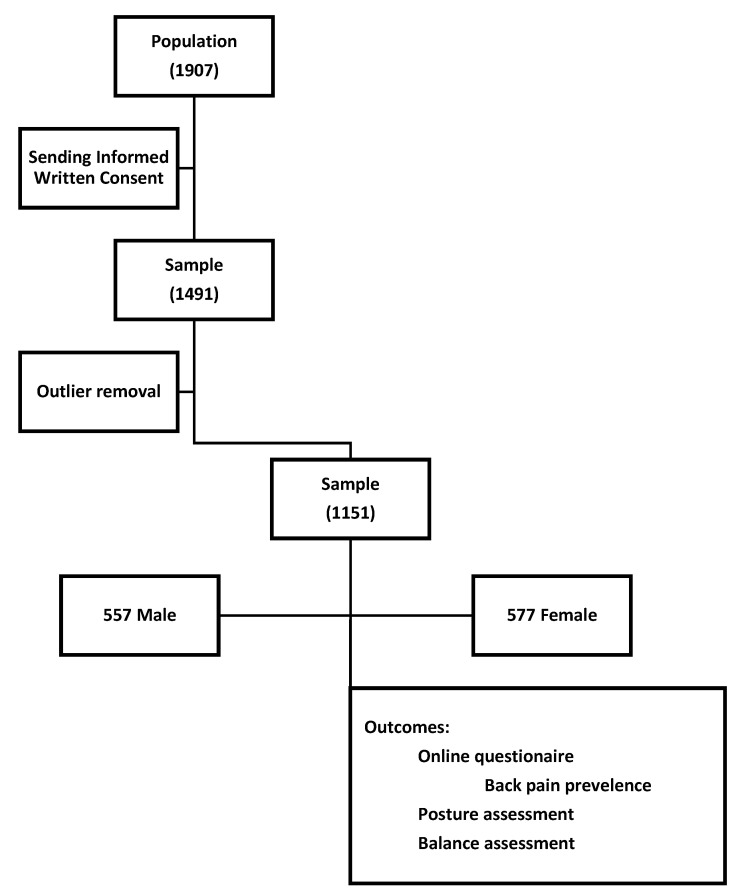
Study design.

The study design is shown in Figure 1.

### 2.1. Instruments

An online questionnaire (Google Forms) was used to characterize the sample in terms of back pain and its severity. The questionnaire included questions about the location of back pain and its occurrence. An 11-item numerical scale (NRS-11) linked to the Face Pain Scale-Revised was used to quantify pain. This instrument is recommended for self-report in children and adolescents, and the combination of the two instruments makes it easier for children to describe their pain [21].

Body mass index (BMI) was determined from the mass and height of the participants.

Postural angle assessment in the spinal regions was performed using the Spinal Mouse^®^ (Idiag, Voletswil, Switzerland). The Spinal Mouse (SM) is a non-invasive mobility device used to quantify posture and spinal mobility. The spinal regions studied were the thoracic spine, lumbar spine, and pelvic region. The cervical spine was not included in the assessment because cervical spine measurements are not valid according to the manufacturer. The software used with SM was IDIAG M360pro^®^ version 7.6 (Idiag, Voletswil, Switzerland). An internal algorithm converts raw measurements into clinically relevant data, namely thoracic kyphosis, lumbar lordosis, and pelvic tilt angles.

The stable static balance evaluation was performed with a pressure platform to obtain the stabilometry parameters. The platform used was the Namrol^®^ Podoprint^®^ printing platform (Medicapteurs France SAS, Balma, France). The overall size of the platform is 610 × 580 × 9 mm for a 400 × 400 mm working surface with 1600 sensors (1 per cm^2^). The software used was Podoprint software (Medicapteurs France SAS, Balma, France).

### 2.2. Posture Assessment

Measurements were performed with the students in the orthostatic reference position and with minimal clothing in the trunk (the girls used adhesive tape to hold their bras, always assisted by the researcher and a female teacher; the boys had the torso without clothes). The assessment was conducted individually to preserve the privacy of each person assessed. Postural analysis in orthostatic position was performed by moving the Spinal Mouse along the spine of the subjects from the 7th cervical vertebra to the 2nd sacral vertebra.

The assessment took place in a room reserved for this purpose, where privacy was maintained and which offered appropriate environmental conditions, especially in terms of temperature (about 22 °C) and brightness. The privacy of the students was always maintained by having a screen-separate place for the analysis. The average duration of each examination was approximately 5 min per participant.

For evaluation of the lumbar and thoracic spine angles, the respective Cobb angles in the sagittal plane are considered the gold standard [22], mainly in children [23].

For the evaluation of the thoracic kyphosis angle, the Cobb angle is measured by drawing a line through the upper endplate of T4 and a second line through the lower endplate of T12 [24]. For the evaluation of the lumbar lordosis angle, the Cobb angle is measured by drawing a line through the upper surface of the first lumbar vertebra and a second line through the surface of the first sacral vertebra [25]. Assessment of the sacrum was performed by pelvic tilt angle in the sagittal plane. The pelvic tilt is measured by the angle between the vertical and the line connecting the center of the upper sacral plate to the hip axis. There is a strong correlation between pelvic morphology and sacrum morphology and pelvic tilt [26]. As mentioned before, the thoracic kyphosis, lumbar lordosis, and pelvic tilt angles were all computed within the IDIAG M360pro^®^ software from Spinal Mouse data and reported by the software [27,28,29].

The reference angles for spinal curvatures in the sagittal plane in healthy children are thoracic kyphosis (33.3 ± 2.4°) and lumbar lordosis L1–L5 (39.6 ± 2.6°). The reference angles for adolescents for the same regions are thoracic kyphosis (35.4 ± 1.9°) and lumbar lordosis L1–L5 (42.7 ± 1.5°) [30]. The reference values for pelvic tilt in children and adolescents are 7.7 ± 8.3° [31].

### 2.3. Balance Assessment

The stable static balance evaluation was performed with a pressure platform to obtain the stabilometry parameters. The data collected were the CoP sway path length (Sway path CoP), CoP ellipse area/surface displacement (Area CoP), CoP mean velocity displacement (v CoP), CoP lateral/medial mean velocity displacement (vML CoP), CoP anterior/posterior mean velocity displacement (vAP CoP), CoP lateral/medial total displacement (dML CoP), and CoP Anterior/Posterior total displacement (dAP CoP).

Children were placed on the print platform for a period of 10 s and were asked to fixate a point in front of the wall. Due to the large sample size and because the subjects were children, we decided to use a shortened analysis period (10 s). This reduced time period has been used in other studies with clinical significance [32]. The assessment was performed with eyes open only.

The balance assessment took place in a separate room from the posture assessment, but this room also provided the environmental and privacy conditions necessary for the comfort of the children and adolescents as well as for the evaluation.

### 2.4. Statistical Analysis

Descriptive statistics were used to characterize the study sample. Normality of conditions was assessed with the Kolmogorov–Smirnov test, and an analysis of outliers was performed for all variables included in the study to remove them from the statistical analysis.

Mann–Whitney U-test was used to estimate differences in the studied variables between the two gender groups (female/male).

The differences between the age groups (children and adolescents) and the studied variables were assessed using the independent-samples Mann–Whitney U-test.

To analyze the correlation between postural angles and stable static balance variables with the anthropometric variables, Pearson’s correlation test was used.

Multiple linear regression was used to test if postural angles in the spine regions significantly predicted stable static balance variables in children and adolescents.

For the association between the manifestation of spinal pain and the variables studied, binary logistic regression was used to calculate the odds ratio. Statistical significance was set at α = 0.05. The software IBM SPSS (IBM Corp, Armonk, NY, USA, version 26) was used.

## 3. Results

The total number of students who were given informed consent after the description of the study was 1907, of whom 1491 agreed to participate in the study, comprising 729 female (48.9%) and 762 male (51.1%).

After analyzing the data, the outliers from the variables included in the study were removed, leaving 1154 individuals in the sample. Of these, 557 (50%) were male, and 577 (50%) were female.

Analyzing the results shown in Table 1, we can note that there are no differences between genders in terms of age (*p*-value > 0.877) and in the dAP CoP (*p*-value > 0.113), but in the other variables studied, these differences are significant. In anthropometric variables, males have higher values in almost all variables studied, except for BMI, where females have higher values. In the stabilometric variables, female individuals have lower values compared to male individuals. This relationship changes when comparing the variables of postural angles of the different regions of the spine, with female individuals showing higher values in all spinal segments studied.

The values for the stabilometric variables are smaller than usual mainly due to the small time used in the evaluation, just 10 s, due to the reasons already mentioned in Section 2.3.

We divided the total sample into age-related groups, namely children and adolescents following Furlanetto et al. [30]. The adults (18 and over in Furlanetto et al. classification) were only 25, and their parameters were indistinguishable statistically from those of the adolescents; therefore, we have merged the adults (25 subjects) into the adolescents’ group. Comparing the studied variables with age-dependent groups (Table 2), the stabilometry values are higher in children compared to adolescents for all studied stabilometry parameters.

In the postural angle of the thoracic spine, children have a lower postural angle than adolescents.

The correlation of the anthropometric variables against the stabilometry variables and the postural angles (Table 3) shows a weak correlation between the variables, although it is statistically significant except for the correlation between lumbar angles and age and weight. The correlations are negative for almost all variables, except for thoracic angles against all anthropometric variables and lumbar angles in their correlation with BMI. All these correlations are small but significant.

Table 4 shows the results of the multiple linear regression in which we tested whether the postural angles of the different regions studied significantly predicted the stabilometry results. A model of the following form was used:

$$Y=C_{0}+B_{1}*\mathrm{Pelvic}+B_{2}*\mathrm{Thoracic}+B_{3}*\mathrm{Lumbar}+\epsilon$$

**Figure 2:**
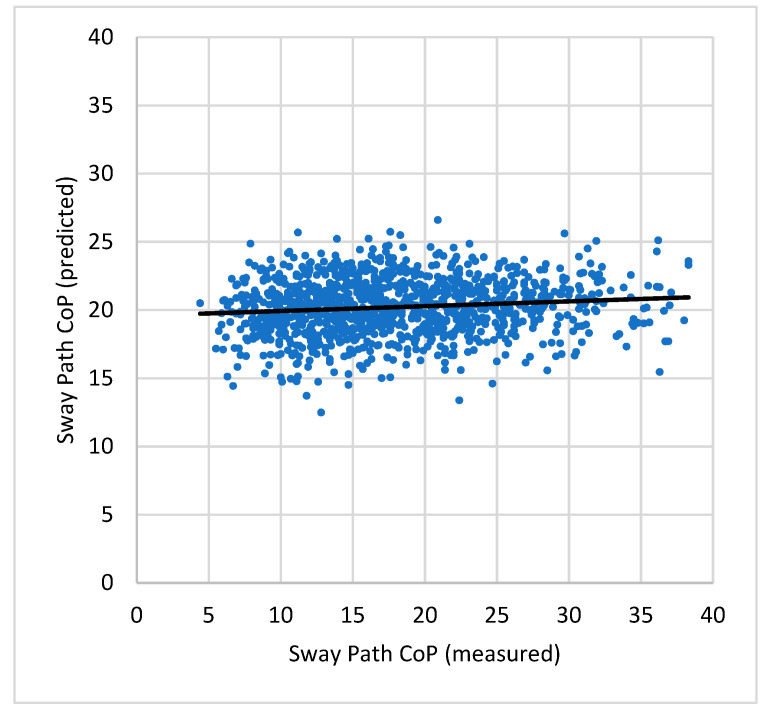
Scatterplot of the predicted (from Table 3 values) over the measured Sway Path CoP. The black line is a linear fit of the plotted points, just to help in the visualization.

When analyzing the results, it was found that the fit of the postural angles to predict the stabilometry values had very low values of the coefficient of determination (R^2^); that is, the fit is very poor. This can be seen in Figure 2, where an example for the Sway path CoP is shown. The coefficients of the fit (*B*_1_, *B*_2_, *B*_3_) also have very small values, implying that the fit is almost just the constant value (*C*_0_), i.e., a horizontal line.

Most of the fits, and most of the fitting coefficients, have statistically significant values. Nevertheless, the fittings are very poor.

In our data, Table 5, binary logistic regression indicates that the angle of thoracic kyphosis is the only significant predictor of back pain in children and adolescents (Chi-Square = 41.49, df = 10 and *p* = 0.001). The other nine variables were not significant against back pain. The postural angle of the thoracic spine explains only 3% of the manifestation of back pain in children and adolescents, but it is a significant relationship. The greater the thoracic kyphosis, the greater the risk of back pain (OR: 1.030; CI 1.011–1.048).

## 4. Discussion

This study had two main objectives, reflected in two study hypotheses. The first objective was to evaluate the relationship between postural angles in the spine regions and stable static balance variables in children and adolescents. Our second objective was to investigate whether postural angles and stable static balance parameters are related to the manifestation of back pain.

For the first study hypothesis, we can conclude from the data analysis that the postural angles of the different regions of the spine, namely the thoracic, lumbar, and pelvic spine, give poor predictions of balance variables. Most of the fits were statistically significant, but all of them have values of R^2^ very close to zero.

For the second hypothesis, through our analysis, we found one statistically significant relationship between the postural angle of the thoracic spine and the manifestation of back pain in children and adolescents. This risk increases with increasing the angle of thoracic kyphosis, although it is relatively small (3% OR). The other variables did not show a statistically significant association with the manifestation of back pain in children and adolescents. Posture is only one factor among the numerous factors associated with back pain in children and adolescents [4,16].

### 4.1. Differences between Genders

The results of this study show something interesting regarding the difference between genders, namely that girls have lower values of the stabilimetry variables at stable static balance than boys. This observation may indicate the higher stability of the girls. This finding is consistent with the studies conducted by Rusek et al. [33] and Ludwig et al. [15]. These observations have also been made in other studies in adults [34,35]; therefore, it will be important for future studies to examine more closely the neuromuscular patterns associated with gender differences.

### 4.2. Differences between Children and Adolescents

The division into age groups was based on Furlanetto et al. [30]. When comparing the results of stabilometry, it can be seen that balance increases with age, with children having a lower balance index than adolescents. These data are consistent with a systematic review that found that adolescents have higher balance scores compared with children [36]. Older children have higher height, which, according to a recent study analyzing anthropometric variables and balance, not only has negative correlation indices with balance variables, as seen in our study, but is also a predominant factor in explaining balance [37,38].

The thoracic kyphosis curvature showed a linear increase with age, which has been confirmed in other studies seeking to understand the development of thoracic curvature with growth [39,40].

### 4.3. Anthropometric Variables, Static Balance, and Posture

The negative relationship between anthropometric variables and stable static balance was observed for all variables analyzed (Table 3). This indicates that the higher the age, weight, height, or BMI, the lower the values of the stabilometry variables, suggesting for better sensorimotor abilities related to balance. Age is the variable with the highest negative correlation; that is, the older the child is, the better their balance is. These results are consistent with the data in Table 2, where a positive relationship was found between age and balance competence.

Results similar to those of height are found for weight and BMI but with lower correlation coefficients, although statistically significant. This fact may also be related to the fact that older young people have more weight. The BMI results are consistent with studies that have found a negative correlation between balance and BMI [41] and also are in line with the results of a recent study in which children and adolescents with higher BMI performed better on balance parameters [33].

The results related to the correlation between the postural variables and the anthropometric variables showed a significant negative correlation between the pelvic tilt and the age, weight, and height, although the correlations are very low. The lumbar lordosis shows a significant correlation with height and BMI. For height, this correlation is negative, and for BMI, it is positive. This correlation is also very low but is consistent with the results of other studies [42]. For the data of the thoracic kyphosis, the correlation is positive for all variables, especially for weight and BMI, with the latter correlation being more significant. Thus, the higher the BMI, the greater the angle of thoracic kyphosis. These data are consistent with some studies highlighting the positive correlation between BMI and hyperkyphosis [43]. Height also showed a positive correlation with the increase in the curvature of thoracic kyphosis, as already underscored in another study [44]. Although the correlation is not as strong as for weight and BMI, it is also significant. This finding may help to better understand the occurrence of hyperkyphosis in children and adolescents.

### 4.4. Posture and Balance

In our study, the postural angles of the three spinal regions studied, namely the pelvic tilt, the lumbar lodosis, and the thoracic kyphosis, have shown to be poor predictors of the stabilometric variables. This predictive relationship, although statistically significant, has very low values of R^2^ for all relationships between variables. These data show a marginal relationship between postural changes and changes in static balance, although other studies have not found a significant correlation between these variables [14,15].

This relationship raises some questions about the normal development of children’s motor skills and posture. A study conducted by Nagymáté et al. [45] concluded that poor posture in children has no clear effect on balance.

Another study investigated the relationship between balance and postural changes in the sagittal plane of the spine and concluded that increases in lumbar lordosis lead to a worsening of the ability to tolerate balance disturbances [46]. Also in our study, the increase in lumbar lordosis was associated with the increase in dML CoP, leading to a decrease in balance, and although statistically significant, it was a very small increase (linear fit coefficient of 0.007).

Another interesting result relates to pelvic tilt and its relationship to balance. Of all the parameters related to postural angles, pelvic tilt is the one most related to balance (although the relationship is small), in this case negative. When the anterior pelvic tilt increases, the stabilometry parameters decrease, suggesting better balance. These results are consistent with those from Mac-Thiong et al. [47] study, which showed that pelvic tilt increases with age, most likely to avoid an insufficient anterior shift of the body’s center of gravity.

### 4.5. Back Pain and Balance Parameters

Among the studies that tried to identify the risk factors that influence back pain in children and adolescents, the use of posture variables is common, but their relationship with a balance is not fully clarified [4,48].

In our study, the variables related to static balance did not contribute to an increase in the probability of having back pain. However, when we analyze the postural angles and their relationship with the manifestation of back pain, this relationship is significant for thoracic kyphosis. The greater the angle of the thoracic kyphosis, the greater the risk of back pain. Although this increment is low, it is significant. There have been several studies addressing back pain and the lumbar lordosis, particularly low back pain [49,50,51], but the association with thoracic spine postural angle as a predictor of back pain has been little studied, except in more severe clinical conditions such as Scheuermann’s disease [52,53].

Although there is no consensus on the risk factors for back pain in children and adolescents, posture seems to be an important factor, especially sitting posture [4,54,55]. In our study, assessment was performed in the upright position, and assessment of posture in this position is also a common clinical practice. These data confirm that clinical posture assessment is an important tool for the early detection of potential risk factors related to posture itself and back pain.

Some other factors that may be related to back pain, such as the time spent using a smartphone or the time spent practicing physical exercises per week, will be the subject of a forthcoming article.

### 4.6. Practical Implications of the Study

Motor skills, especially balance in children, are essential for normal musculoskeletal development. Postural changes are increasingly evident in today’s society, where sedentary lifestyles and poor posture are on the rise. This study confirms the relationship between posture and balance. Although it is a weak relationship, it is significant. Therefore, we must work with schools and teachers to promote the importance of physical activity and exercise in physical education classes, where balance is a modality of increasing importance. This promotion must also include work on posture correction in the classroom so that the results related to prevention are more effective and sustainable.

### 4.7. Limitations of the Study

One of the principal limitations of this study is the short time used for the stabilometric evaluation, just 10 s. We selected this value due to the number of subjects to evaluate and the fact that a good fraction of them was very young, and it was difficult for them to stand still for longer periods. We believe this was the main cause of the lower than usual values for the stabilometric variables.

Furthermore, this study has natural limitations characteristic of cross-sectional studies in understanding a phenomenon as complex as human balance and its relationship to spinal posture. Thus, although we can establish relationships between the parameters studied, we cannot establish direct causality between them in children and adolescents.

It would be interesting to add other measurement tools, such as surface EMG, to analyze the muscle activity of the muscles involved in postural control, but the large sample size and the younger population (due to the characteristics of the children) would require a rigorous and rapid process of data collection, something not easy to apply in practice.

Despite these limitations, we were able to contribute a little more to the understanding of the already complex relationship between balance and posture in the younger population.

## 5. Conclusions

With this work, we contribute to a more comprehensive understanding of the relationship between spinal postural angles and static balance in children and adolescents. Postural changes in children and adolescents and the consequences of inefficient balance are becoming increasingly important in developing programs to prevent musculoskeletal pathologies in today’s children.

## Figures and Tables

**Table 1:** Sample characterization, stabilometric and angular variables, separated by gender. Comparison between genders for all variables.

	Female	Male	*p*-Value *
	Mean/SD	Mean/SD	
Age (year)	14.16/2.29	14.16/2.26	0.877
Mass (kg)	54.53/11.59	57.33/13.29	<0.001
Height (cm)	158.23/8.53	165.27/13.28	0.000
BMI (kg/m^2^)	21.65/3.50	20.77/3.11	<0.001
Sway path CoP(mm)	17.02/6.87	18.85/6.76	<0.001
Area CoP (mm^2^)	10.35/8.89	11.29/8.23	0.001
v CoP (mm/s)	1.57/0.64	1.74/0.63	<0.001
vML CoP (mm/s)	1.18/0.51	1.32/0.52	<0.001
vAP CoP (mm/s)	1.02/0.44	1.11/0.42	<0.001
dML CoP (mm)	0.82/0.43	0.89/0.42	0.003
dAP CoP (mm)	0.92/0.49	0.96/0.48	0.113
Pelvic tilt (°)	20.45/6.09	15.25/5.48	0.000
Lumbar lordosis (°)	35.05/7.73	27.66/7.49	<0.001
Thoracic kyphosis (°)	47.32/9.78	45.31/8.50	0.000

* Mann–Whitney U-test: (level of significance 95%).

**Table 2:** Sample characterization, stabilometric and angular variables, separated by groups: children and adolescents.

Age (Year)	9–11	12–19	*p*-Value *
Number of Subjects	195	959	
	Mean/SD	Mean/SD	
Mass (kg)	42.92/10.40	58.57/11.23	0.000
Height (cm)	146.98/8.36	164.75/9.85	0.000
BMI (kg/m^2^)	19.64/3.36	21.53/3.24	<0.001
Sway path CoP(mm)	21.83/6.42	17.14/6.69	0.000
Area CoP (mm^2^)	14.94/9.38	9.98/8.16	<0.001
v CoP (mm/s)	2.01/0.60	1.58/0.62	0.000
vML CoP (mm/s)	1.52/0.52	1.19/0.50	0.001
vAP CoP (mm/s)	1.29/0.41	1.02/0.42	0.001
dML CoP (mm)	1.01/0.43	0.82/0.42	<0.001
dAP CoP (mm)	1.13/0.51	0.90/0.47	<0.001
Pelvic tilt (°)	17.12/4.81	18.00/6.61	0.075
Lumbar lordosis (°)	30.64/7.76	31.50/8.59	0.222
Thoracic kyphosis (°)	44.42/9.69	46.70/9.07	0.002

* Independent-samples Mann–Whitney U-test (level of significance 95%).

**Table 3:** Pearson correlation between anthropometric variables and stabilometry and angular variables.

	Age	Weight	Height	BMI
Sway path CoP	−0.238 **	−0.163 **	−0.164 **	−0.111 **
Area CoP	−0.292 **	−0.139 **	−0.147 **	−0.082 **
v CoP	−0.287 **	−0.158 **	−0.160 **	−0.107 **
vML CoP	−0.279 **	−0.162 **	−0.158 **	−0.115 **
vAP CoP	−0.265 **	−0.137 **	−0.145 **	−0.087 **
dML CoP	−0.190 **	−0.135 **	−0.108 **	−0.108 **
dAP CoP	−0.191 **	−0.108 **	−0.125 **	−0.053 **
Pelvic tilt	0.077 **	−0.092 **	−0.130 **	−0.003
Lumbar lordosis	0.053	−0.010	−0.108 **	0.102 **
Thoracic kyphosis	0.123 **	0.307 **	0.136 **	0.339 **

** The correlation is significant at the 0.01 level.

**Table 4:** Multiple linear regression between the angles of sagittal spinal posture and the stabilometric variables.

	**B**	**95% CI**	**β**	***t***	***p*** **-Value**
**Sway Path CoP** **(R^2^ = 0.03, F (3, 1150) = 10.36, *p* = <0.001)**					
**Constant**	23.591	21.207, 25.975		19.414	<0.001
**Pelvic tilt**	−0.226	−0.356, −0.097	−0.209	−3.425	<0.001
**Lumbar lordosis**	0.069	−0.038, 0.175	0.085	1.270	0.204
**Thoracic kyphosis**	−0.081	−0.140, −0.023	−0.109	−2.713	0.007
**Area CoP** **(R^2^ = 0.01, F (3, 1150) = 3.18, *p* = 0.023)**					
**Constant**	14.623	11.621, 17.624		9.559	<0.001
**Pelvic tilt**	−0.171	−0.334, −0.008	−0.127	−2.056	0.040
**Lumbar lordosis**	0.059	−0.075, 0.193	0.058	0.863	0.388
**Thoracic kyphosis**	−0.056	−0.130, 0.18	−0.060	−1.480	0.139
**v CoP** **(R^2^ = 0.03, F (3, 1150) = 10.31, *p* < 0.001)**					
**Constant**	2.178	1.956, 2.400		19.258	<0.001
**Pelvic tilt**	−0.021	−0.033, −0.009	−0.211	−3.454	<0.001
**Lumbar lordosis**	0.007	−0.003, 0.016	0.087	1.301	0.194
**Thoracic kyphosis**	−0.008	−0.013, −0.002	−0.109	−2.698	0.007
**vML CoP** **(R^2^ = 0.02, F (3, 1150) = 8.96, *p* < 0.001)**					
**Constant**	1.657	1.476, 1.837		17.992	<0.001
**Pelvic tilt**	−0.018	−0.027, −0.008	−0.215	−3.513	<0.001
**Lumbar lordosis**	0.007	−0.001, 0.015	0.111	1.657	0.098
**Thoracic kyphosis**	−0.007	−0.011, −0.002	−0.117	−2.901	0.004
**vAP CoP** **(R^2^ = 0.02, F (3, 1150) = 8.99, *p* < 0.001)**					
**Constant**	1.387	1.236, 1.538		18.053	<0.001
**Pelvic tilt**	−0.013	−0.021, −0.004	−0.185	−3.035	0.002
**Lumbar lordosis**	0.003	−0.004, 0.010	0.063	0.937	0.349
**Thoracic kyphosis**	−0.004	−0.008, −0.001	−0.091	−2.259	0.024
**dML CoP** **(R^2^ = 0.01, F (3, 1150) = 3.68, *p* = 0.012)**					
**Constant**	1.070	0.921, 1.218		14.098	<0.001
**Pelvic tilt**	−0.011	−0.019, −0.003	−0.168	−2.737	0.006
**Lumbar lordosis**	0.007	0.000, 0.014	0.137	2.033	0.042
**Thoracic kyphosis**	−0.005	−0.009, −0.001	−0.108	−2.665	0.008
**dAP CoP** **(R^2^ = 0.004, F (3, 1150) = 1.46, *p* = 0.223)**					
**Constant**	1.095	0.925, 1.265		12.644	<0.001
**Pelvic tilt**	−0.003	−0.012, 0.006	−0.039	−0.629	0.529
**Lumbar lordosis**	−0.001	−0.008, 0.007	−0.012	−0.185	0.853
**Thoracic kyphosis**	−0.002	−0.006, 0.002	−0.033	−0.808	0.419

**Table 5:** Manifestation of back pain against balance and posture parameters.

	Odds Ratio	95% CI	*p*-Value
Sway path CoP	1.026	0.760/1.385	0.867
Area CoP	0.988	0.951/1.026	0.520
v CoP	0.644	0.028/14.819	0.783
vML CoP	0.748	0.077/7.273	0.802
vAP CoP	1.186	0.152/9.276	0.871
dML CoP	1.381	0.827/2.308	0.217
dAP CoP	0.901	0.578/1.404	0.644
Pelvic tilt	1.027	0.987/1.069	0.184
Lumbar lordosis	0.997	0.965/1.030	0.854
Thoracic kyphosis	1.030	1.011/1.048	0.002

## Data Availability

The data underlying this study are available from the corresponding author upon request.
